# Integrated early childhood development services improve mothers’ experiences with prevention of mother to child transmission (PMTCT) programs in Malawi: a qualitative study

**DOI:** 10.1186/s12913-021-06342-2

**Published:** 2021-04-15

**Authors:** Kathryn Dovel, Pericles Kalande, Evelyn Udedi, Tijana Temelkovska, Julie Hubbard, Chipariro Mbalanga, Laurie Bruns, Siyenunu Mulungu, Sundeep Gupta, Linda Richter, Thomas J. Coates

**Affiliations:** 1 Partners in Hope, Lilongwe, Malawi; 2 Division of Infectious Diseases, David Geffen School of Medicine, University of California Los Angeles, Los Angeles, CA USA; 3 David Geffen School of Medicine, University of California Los Angeles, Los Angeles, USA; 4 University of California Global Health Institute, San Francisco, USA; 5 DSI-NRF Centre of Excellence in Human Development, University of the Witwatersrand, Johannesburg, South Africa

## Abstract

**Background:**

HIV-positive mothers who face the dual burden of HIV-positive status and motherhood, may benefit from holistic services that include early childhood development (ECD). We evaluated the acceptability and impact of integrated ECD-PMTCT interventions for mothers and their children.

**Methods:**

We implemented an integrated ECD-PMTCT intervention in 4 health facilities in Malawi for HIV-positive mothers and their infants. WHO/UNICEF Care for Child Development (CCD) education and counseling sessions were offered during routine PMTCT visits between infant age 1.5–24 months. From June–July 2019, we conducted in-depth interviews with 29 mothers enrolled in the intervention for ≥6 months across 4 health facilities. The interview guide focused on perceived impact of the intervention on mothers’ ECD and PMTCT practices, including barriers and facilitators, and unmet needs related to the program. Data were coded and analyzed using constant comparison methods in Atlas ti.8.

**Results:**

The vast majority of mothers believed the ECD-PMTCT intervention improved their overall experience with the PMTCT services, strengthened their relationship with providers, and excited and motivated them to attend PMTCT services during the postpartum period. Unlike prior experience, mothers felt more welcome at the health facility, and looked forward to the next visit in order to interact with other mothers and learn new ECD skills. Mothers formed new social support networks with other mothers engaged in ECD sessions, and they provided emotional and financial support to one another, including encouragement regarding ART adherence. Mothers believed their infants reached developmental milestones faster compared to non-intervention children they observed at the same age, and they experienced improved engagement in caregiving activities among male caregivers. Nearly half of women requested additional support with depression or anxiety, coping mechanisms to deal with the stresses of life, or support in building positive dynamics with their male partner.

**Conclusion:**

The integrated ECD-PMTCT intervention improved mother’s experiences with PMTCT programs and health care providers, increased ECD practices such as responsive and stimulating parenting, and created social support networks for women with other PMTCT clients.

**Supplementary Information:**

The online version contains supplementary material available at 10.1186/s12913-021-06342-2.

## Background

Sub-Saharan Africa (SSA) is home to 87% of all HIV infected mothers and children worldwide [1]. HIV exposed children are at significantly greater risk of developmental delays than unexposed children, independent of their final HIV status [2, 3]. Mothers living with HIV face challenges including the dual burden of living with HIV and newly entering into motherhood, which can lead to substantial loss to follow up from HIV treatment programs [4]. Due to increased vulnerabilities, mothers living with HIV and their infants may especially benefit from holistic services aimed at improving their and their child’s overall wellbeing, such as early childhood development services.

Early childhood development (ECD) within the first 5 years of life is foundational to an individual’s development, wellbeing, and potential capital throughout the life course [5]. ECD services comprise a package of comprehensive, evidence-based interventions aimed at addressing child health holistically and providing children with an optimal start in life. Nurturing Care, defined as the policies, programs and services that enable caregivers and communities to ensure childhood wellbeing and learning, was recently formalized through the development of WHO’s Nurturing Care Framework [6].

In the past decade, there has been increasing recognition of the importance of ECD, with many countries in sub-Saharan Africa (SSA) adopting national ECD policies [7]. As ECD interventions expand, the most scalable models have been integrated into existing health systems that already reach families and infants and young children, such as breastfeeding promotion campaigns, vaccination campaigns, and nutrition promotion efforts [8, 9]. However, to our knowledge, ECD interventions have yet to be routinely integrated into PMTCT settings or target HIV affected children, who are among the most vulnerable to poor developmental outcomes.

Prevention of mother-to-child transmission (PMTCT) programs provide a strong, developed infrastructure for reaching nearly all HIV-positive mothers and their infants in SSA. PMTCT strategies have been very effective in reducing HIV transmission to infants [1]. Approximately 90–95% of HIV positive pregnant women initiate or are already on antiretroviral therapy (ART) during pregnancy [10], making PMTCT services an ideal entry point for integrated ECD services for HIV- affected children.

Integrated ECD-PMTCT interventions have several potential benefits. First, ECD interventions have been shown to have the greatest benefit amongst vulnerable or disadvantaged children [11]. ECD strategies incorporated into existing PMTCT programs may improve early childhood development among children affected by HIV, providing long term benefits in terms of health, wellbeing and economic attainment. Second, integrated ECD-PMTCT services may provide the additional support and holistic care needed to enhance women’s experiences with PMTCT services and promote ART retention among mothers [12]. However, to our knowledge only one study has piloted an integrated ECD-PMTCT intervention [13].

We used qualitative data from an integrated ECD-PMTCT intervention in Malawi to provide insights into the percieved impact of the intervention among participating women, examining percieved impact on ECD among their youngest child (primary outcome) and women’s experience with and engagemnet in the PMTCT program (secondary outcome). In addition, we examine remaining unment needs for women attending the ECD-PMTCT intervention.

## Methods

### The intervention

Mothers and their young children (aged 0–8 weeks) were recruited in four health facilities in central Malawi to participate in an integrated ECD-PMTCT intervention. Mothers were eligible if they were HIV-positive, enrolled in the PMTCT program at a participating facility, and had a child < 8 weeks of age. Facilities selected for the intervention varied in size, type (district hospital, mission hospital, health center), and district (see Table 1). The intervention provided interactive ECD skills development sessions during PMTCT wait times and thus did not require additional time or resources from participants. ECD sessions corresponded with mothers’ ART refill schedules, which were typically every 1–3 months depending on the duration of ART prescribed, length of time mothers had been on ART, and mothers’ overall adherence. Mothers were also permitted to attend ECD sessions any other time they attended the health facility; however, this was less common.

**Table 1:** Facility Characteristics

Facility Name	District	Type of Facility	Number of Women Enrolled in ECD-PMTCT Program
Daeyang Luke Hospital	Lilongwe	Mission Hospital	105
Malingunde Health Center	Lilongwe	Health Center	55
Kasungu District Hospital	Kasungu	District Hospital	219
Nkhotakota District Hopsital	Nkhotakota	District Hospital	119

Monthly ECD sessions completed during PMTCT waiting times were informed by the WHO-UNICEF “Care for Child Development” (CCD) package [14] that provides information and coaching on the cognitive, emotional and communication needs of infants. Main content covered by the CCD includes stimulation, responsive parenting, early learning and education, and play and communication. Content was delivered through simple interactive play lessons and focused on the following components: (1) who is a caregiver, (2) roles of the caregiver, (3) nurturing care, (4) health and brain development, (5) play in early years, and (6) supporting language and literacy in 0–3 years. Mothers were sensitized to the importance of their responsiveness to their young child, including eye-contact, vocalizations and imitation with their infants. They were also taught to identify and respond to developmental milestones appropriate for their infants’ age. All sessions were implemented by “Expert Clients (EC)”, a Malawi cadre of HIV-positive volunteer community members who provide HIV counseling and support to HIV-positive individuals [15]. Two ECs at each facility received 2 weeks training in ECD and CCD and were responsible for implementation of the ECD sessions at their facilities. After 24 months, mothers and their infants transition to non-PMTCT care and thus complete the program.

### Data collection

In-depth interviews were conducted from June to July 2019 with a random subset of mothers enrolled in the ECD-PMTCT program for ≥6 months in order to ensure mothers had the opportunity to be exposed to most of the ECD curriculum from CCD package. We selected a random sample of mothers in order to have a representative description of women’s perceptions of the program. – this also ensured there were no biases in sample selection, such as selecting women who were actively engaged in the program or known to think positively about the program. Program enrollment materials were digitized and 32 women were randomly selected by the data manager using a computer-generated random sequence of numbers. Random selection was stratified by health facility to ensure all facilities were represented since implementation and acceptability of the intervention may depend heavily on facility-level factors, such as personnel and characteristics of the community served (8 women per health facility, with 4 health facilities in total), as well as by duration on ART. At each facility, 50% of the randomly selected mothers had initiated ART ≤12 months ago and 50% ≥ 12 months ago since newly diagonized mothers may react differently with HIV programs and their infants. Selected women were contacted by the study staff and invited to attend the heatlh facility for an in-depth interview.

The interview guide and analysis framework were developed based on existing literature [7, 12, 13] and previous experiences with success' and challenges with the ECT-PMTCT program to date. The aim of the interviews were to assess acceptability of the program, perceived benefits of the program, and remaining unmet needs that were not addressed in the program. The guide was focused on three primary domains: (1) experiences with the ECD-PMTCT program, including what was learned, attendance, favorite (and least favorite) sessions, and relationships built with other mothers (2) perceived impact of the sessions, including how ECD education was applied within caregiving practices both themselves and jointly with their male partner and (3) unmet needs of mothers during the program. Guides were piloted amongst three women to ensure comprehensibility, and were then further refined based on feedback. See Additional file 1 for the final in-depth interview guide in English.

All interviews were conducted at a participating health facility by a trained female research assistant in the local language (Chichewa), and ranged in duration from 30 to 50 min. Participants were compensated 4000 Malawi Kwacha (approximately US $5) for their transportation costs to attend the interview.

### Data analysis

Audio recordings were translated and transcribed into English for analysis. A preliminary codebook was generated using deductive coding from existing literature and inductive coding from pilot interviews and novel findings from early transcripts. Two investigators (TT and PK) coded the same five transcripts separately using Atlas.ti (version 1.6). Coding was reviewed with KD and differences in coding were resolved, creating a finalized codebook and code definitions. An additional two transcripts were simultaneously coded, with similar codes between investigators. TT and PK coded all remaining transcripts using the finalized codebook. Data were analyzed using constant comparison methods. In this paper we present dominant themes related to mothers’ perceived benefits of the program and remaining unmet needs.

### Ethical approval

Ethical approval was attained from University of California Los Angeles (UCLA) and the Malawi institutional review board, National Health Sciences Review Committee (NHSRC). The datasets used and/or analysed during the current study are available from the corresponding author on reasonable request.

## Results

Of the 29 mothers interviewed, nearly all were married. Women had a mean of 4 children (range = 1 to 8 children) and mean age of 30 years (range = 18 to 38 years). Half reported informal employment or entrepreneurial activities as the primary household income, with only two participants reporting any formal employment within the household (either for themselves or their husbands). A little less than half of mothers had initiated ART within the past year. The vast majority of mothers attended ECD sessions every month and only one mother stopped attending the ECD-PMTCT program completely (both ART and ECD services). See Table 2.

**Table 2:** Demographic characteristics of participants

Variable	Participants (***n*** = 29)
Age, mean (range)	29.8 (18–38)
Married, n (%)	20 (69.0)
Number of children, median (range)	4 (1–8)
Household primary income, n (%)	
Formal employment	0
Informal employment	29 (100)
Not working	0
Education, n (%)	
No education	3 (10.3)
Primary	12 (41.4)
Secondary or higher	14 (48.3)
Male partner involvement, n (%)	
With infant every day	11 (37.9)
With infant several days a week	10 (34.5)
With infant < 1 days a week	1 (3.5)
Not involved at all	7 (24.1)
Discuss PMTCT-ECD program with male partner, n (%)	21 (72.4)
Length of time on ART, n (%)	
< 12 months	13 (44.8)
> 12 months	16 (55.2)
ECD session attendance, n (%)	
Attend most sessions	28 (96.5)
Attend some sessions	1 (3.5)
Dropped out of program	0

### Impact of ECD-PMTCT program

Nearly all mothers reported that they enjoyed being in the ECD-PMTCT program and prioritized program attendance. Women would ensure they arrived at the PMTCT clinic early in order to attend ECD sessions, and actively engaged in the ECD lessons presented.

*“I am satisfied with everything that is why even when I am late, I make sure to meet with the ECD counsellors.” Mother of 2, age 25–29*

*“My experiences in the ECD program is all good, right now my child is growing up with health [y] and knowledge, I did not know that such things can happen.” Mother of 5, age 30–34*

#### ECD benefits

Most mothers believed the integrated ECD-PMTCT program was beneficial for their children and was critical to their own development as caregivers. Mothers identified several tangible ECD-related benefits of being part of the program, including improved parenting techniques (i.e. use of toys, understanding developmental milestones, and responsive parenting) and observed perceived advanced development among their children. Mothers who had multiple children reflected on how their children who participated in the ECD-PMTCT program were more interactive and engaged (i.e. using facial expressions, development of fine motor skills, vocalizing) when compared with older siblings when they were the same age, and they attributed these changes to the ECD program.

*“Yes, there are changes, because all my children did not start walking as quickly as my youngest, he started walking with 9 months old. This is because of the program as I was able to teach him everything.” Mother of 4, age 30–34*

*“When my child was six weeks old I started having [ECD] lessons. They told me to make toys, for example a ball, doll and a car, and to call the child’s name and friends’ names. I started seeing change when the child reached five months old. … I will continue to follow what I learn at the hospital because its good. “ Mother of 8, age 30 -34*

Women also believed the integrated ECD-PMTCT program improved their male partners' parenting behavior and improved their overall experience as joint caregivers. Of the mothers who reported being in a relationship with the father of their youngest child, the majority reported that since starting the program, the father had increased the time spent with the child, and that he was now implementing ECD lessons learned from the program. Nearly half of mothers in a relationship with the father reported improved communication between the mother and father, attributing this change to having a joint purpose around ECD for their children and responsive parenting.

*“After I told him about ECD, he is active with the child. He makes him porridge, sings to him and plays with him. When the child sees his father, he makes noise indicating that he has seen him.” Mother of 1, age 20–24*

*“Me and my child we are living in harmony because we are raising this child together and the child of the father has just realized that the responsibility is for the both of us.” Mother of 4, age 30–34*

#### PMTCT benefits

Mothers cited several benefits of their experiences with PMTCT due to the integrated ECD-PMTCT program. Women believed that having ECD sessions during the PMTCT waiting period improved their overall experience with the ART program and with the health system more broadly. ECD sessions provided additional motivation for attendance and excitement for learning something new as opposed to simply collecting ART medication.

“*At first we just came and got assisted and left; we did not care about anything else apart from just getting medical help. But now with ECD I am interested in knowing about what I am going to learn about my child through ECD.” Mother of 2, age 35–39*

*“The change is that even when a nurse is alone, we don’t get tired of waiting as we are occupied with the ECD activities, whilst in the past before the program, we thought the happenings [waiting times] were just time consuming.” Mother of 4, age 30–34*

ECD sessions were also used to form social support networks with other women living with HIV. Mothers provided each other with support and advice regarding everyday life situations, such as emotional encouragement and financial assistance when needed.

*“We ask about how their child is or whether they practice the things we have learned on their children … . My friend came here at the hospital for ECD and she told me that she did not have any money on her to help her with the hospital fee, so I gave her the money because in the future I may be in a similar situation and I can be able to be assisted by her as well.” Mother of 1, age 20–24*

*“When you are with the fellow women you are sometimes enlightened … Because you learn a lot from each other. “Mother of 3, age 25-29*

Newly founded social networks also encouraged PMTCT activities, as well as encouraged mothers to overcome challenges associated with living with HIV.

*“We encourage each other about life. Other people are shy, others are afraid that when they have been found with HIV then that is the end of their life not realizing that it is a beginning of another life. We encourage each other about that, that when you accept your status you are able to live a healthy life and that your child can also grow a healthy life and that it’s also possible for the child to be born without HIV as long as the mother follows what the doctor is saying.” Mother of 2, age 25–29*

Over half of all women reported that facility staff treated them more positively since they joined the integrated ECD-PMTCT program, and that they felt more welcome at the health facility. Among these mothers, most believed client-provider relationships changed because facility staff had taken time to get to know them. They now talked casually and in detail about their personal lives, whereas prior to the intervention client-provider interactions consisted primarily of drug distribution.

*“What has changed is when you come here you are able to talk freely to the nurse about your problems.” Mother of 2, age 35–39*

*“Because of ECD things have changed because even the nurses or patient attendants, we live with them in harmony, unlike if we were just coming here to get drugs and go home. Now we are able to know them by name and talk to them.” Mother of 2, age 30–34*

#### Recommendations/unmet needs

Despite valuing the integrated ECD-PMTCT program, over half of the interviewed women reported unmet needs that they would like the program to address. Almost half desired specific sessions to help them cope as mothers living with HIV (ie. caregiver wellbeing), including stress management and marital advice. While women did not directly mentioned post-partum depression, several discussed being sad or overwhelmed with their life circumstances.

*“We should also be told as women [how] to be happy; something [strategies] that can make us less stressful and have no worries … Teach us to do away with stress and worries.” Mother of 5, age 25–29*

*“Yes, that we would wish is to teach us how to [financially] take care of our families and [communicate] with husbands admirably. This program would be so helpful then.“ Mother of 4, age 30-34*

Nearly all women reported the need to learn more about nutrition for both themselves and their infants.

*“I would have loved to learn the sort of nutritious food stuffs that we should be giving our children when they reach six months old.“ Mother of 6, age 35-39*

The majority of mothers reflected that talking or playing with infants was culturally uncommon, and the benefits of such activities was not intuitive. Participants believed that the program should have explicit, repetitive lessons on why these activities are needed, and how to talk/play with infants.

*“Other women consider playing with their child as not important because they think it is too time consuming to suspend what they are doing and play with a child. They think that to make dolls for their child to play with is a waste of time.” Mother of 4, age 30–34*

*“They say that small babies can’t hear anything when you’re chatting with them” Mother of 1, age 20–24*

## Discussion

We conducted a qualitative assessment of participants’ perceived impact of an integrated ECD-PMTCT program in Malawi among postpartum mothers living with HIV and their young children. Women believed the intervention had a large impact on both their own experience with PMTCT services, and their infants’ healthy growth and development, along with father’s improved engagement with the childs’ well being and developmental growth. Overall, women prioritized attendance to the program, but still remained with unmet needs such as counseling and mental heatlh support for women who faced multiple stresses in their daily life.

To our knowledge, this is only the second study to examine an integrated ECD-PMTCT program [13]. Women’s improved experiences with PMTCT services is a notable finding, as literature on PMTCT retention show that mothers’ motivation for PMTCT may decline after childbirth, particularly if they are otherwise healthy [16]. Routine ART services are time consuming and perceived to be boring [17], and as a result, there may be little motivation for clinic attendance besides adherence to ART treatment [18]. By integrating ECD sessions into PMTCT waiting periods, women were provided a new purpose for PMTCT attendance beyond ART collection, and described a sense of excitement for clinic visits. The integrated ECD-PMTCT program seemed to provide a community, as well as additional skillsets, that sufficiently motivated mothers to remain engaged in PMTCT services, while potentially impacting child health and development without the cost of additional diagnostics or therapeutic commodities. This is a notable feature that differs from traditional ECD programs that require more expensive interventions (i.e. one-on-one therapy, or nutrition therapy) and warrants further analysis of future ECD-PMTCT integrated models, including costing.

Notably, mothers believed the ECD-PMTCT program improved their relationships with nurses and faciltiy staff. Negative patient-provider interactions have been documented in other qualitative studies to have profound adverse impact on maternal perception of care and subsequent adherence to ART [19, 20]. Mothers praised the integrated, holistic nature of the ECD-PMTCT program and believed their relationship with providers improved because they were able to build relationships with providers through more holistic, humanized interactions during which they discussed issues related to family and children, not only ART drug distribution. While it was hypothesized that the existing strength of the PMTCT infastructire in SSA would provide an ideal integration point for ECD, these unanticipated findings reveal that ECD programs may be of reciprocal value to PMTCT programs by strengthening patient/provider relationships which play a critical role in retention to care.

Participating mothers believed the ECD-PMTCT program resulted in new social support networks. Within the ECD-PMTCT intervention, women built personal relationships and were able to receive and give support on motherhood, family issues, their HIV-positive status, and adherence to the PMTCT program. Other studies find that social support is directly linked with maternal and infant health outcomes [21, 22] and is a primary factor contributing to ART retention and adherence [23, 24]. Further research is needed to understand the longevity of the newly founded social support network once the ECD intervention is completed, and to identify key strategies to promote social bonds among postpartum PMTCT clients.

ECD programs and their impacts have historically focused on women and children, not men. However, literature indicates that male involvement in ECD can result in improved infant feeding practices [25], increased maternal adherence to ART [26, 27], and decreased risk of HIV transmission and child mortality [28]. Although our intervention did not specifically target men, many women reported improved responsive caregiving and participation in childrearing activities by fathers. Some women also reported improved communication with male partners which they attributed to the intervention – this is similar to other literature that shows caregiver-focused interventions can improve family cohesion [29]. Potential engagement in ECD by male partners is encouraging, and underscore the potential interest and missed opportunities to further engage men in ECD interventions. Future ECD programs should develop strategies to directly engage fathers in the intervention.

More than half of participants identified unmet needs related implementing ECD and engaging in PMTCT programs, despite their engagement in the intervention. Mothers requested additional counseling and support for their overall mental wellbeing, including strategies to cope with stress, worry, and how to promote positive relationships between themselves and their male partner. Other studies show that postpartum women living with HIV are at high risk of depression, anxiety, and social tensions [30–33]. Such mental health conditions have been linked with poor ART adherence and retention in some settings [32, 34]. Additional strategies should be incorporated into integrated ECD-PMTCT programs to address depression and social tensions experienced among postpartum women living with HIV.

### Limitations

 We rely on self-reports from mothers who were actively engaged in the ECD-PMTCT program. Further research is needed with mothers who stop attending either ECD sessions or both ECD and PMTCT sessions in order to further understand unmet needs and barriers to program participation. Second, the study is limited to mothers in central Malawi and may not be representative of other settings, although the structure and barriers related to PMTCT programs are similar throughout the region [35], suggesting an integrated ECD-PMTCT program may also work in these settings. Third, our data rely on mothers who were active in the ECD-PMTCT program for a minimum of 6 months. Future analyses should examine if the perceived positive impacts of the integrated program remain salient after the program ends, specifically whether women maintain the newly created social support networks, and the improved interactions with health care workers are sustained over time).

## Conclusions

An integrated ECD-PMTCT intervention in Malawi had high perceived impact among postpartum mothers living with HIV. ECD services offered during PMTCT waiting times were perceived to improve women’s experiences with the PMTCT service and patient-provider interactions. Women also believed that their infants demonstrated improved development and that their male partners became more engaged as a result of the program. Similar interventions hold promise for a double benefit for both ECD outcomes for infants and improved PMTCT engagement for mothers.

## Supplementary Information

**Additional file 1.** Interview guide.

## Data Availability

All data relevant to the study are included in the article.

## Abbreviations

AIDS: Acquired immunodeficiency syndrome
ART: Antriretroviral therapy
CCD: Care for Child Development
EC: Expert client
ECD: Early childhood development
HIV: Human immunodeficiency virus
PMTCT: Prevention of mother to child transmission
