# Supplementary material for: Integrated early childhood development services improve mothers’ experiences with prevention of mother to child transmission (PMTCT) programs in Malawi: a qualitative study
Source: BMC Health Serv Res. 2021 Apr 15;21:348. doi: 10.1186/s12913-021-06342-2 (PMC8048322; doi:10.1186/s12913-021-06342-2)
Supplement: Supplementary file 1 — Additional file 1. Interview guide. [file 12913_2021_6342_MOESM1_ESM.docx]

**IN-DEPTH INTERVIEW FOR HILTON PARTICIPANTS**

**Section 1: Demographics**

**SHORT ANSWER QUESTIONS:** ALL QUESTIONS IN SECTION 1 SHOULD BE ASKED EXACTLY AS WRITTEN. These should be recorded – no need to write down.

| **RA: RECORD UNIQUE ID NUMBER & ECD ENROLLMENT DATE**  1. What is your current age in years? |
| --- |
| 2. How many living children do you have, including children you are caring for as if they are your own? |
| 3. Have you ever attended formal education? |
| 3a. What is the highest year of school you attended?  Example: Standard 1 or Form 3 |
| 4. Are you married or do you have a boyfriend? |
| 5. How long have you been in this relationship? |
| 6. Think about your household. What is the main way your household earns money? |
| 7. Do you do anything else to earn money? What? |

**Section 2: Previous Experiences**

**Experiences with ECD Sessions**

I want to understand what exactly happens when you attend MIP clinic.

1. Think about your usual MIP service that you attend. What do you usually do while you are waiting for the provider?

Do you usually participate in an ECD session on MIP day?

***PROBE, if yes:*** *Why do you make sure to attend the sessions?*

***PROBE, if no:*** *What prevents you from attending the sessions?*

Now I want to understand your experiences with ECD program specifically. I do not work with the ECD program, so please feel free to be open and honest, you can say anything about the program. Everything you say is confidential.

9. Do you do anything else to earn money? What?

Can you please describe for me your overall experience with the ECD program? Is it good or bad? Why?

1. Can you please describe what usually happens during the ECD sessions?
2. What do you learn about during the sessions?

***PROBE:*** *(any topics not mentioned)*

10. Think about the topics you’ve been learning about at the ECD sessions.

- 1. What was the most important thing you learned about? Why?
  2. What was the least important thing you learned? Why?

**(If no least important mentioned, ask about other mothers)**

- 1. What else do you want to learn from the sessions? Do you have any other questions about ECD that have not been answered by the sessions?

Probe: **If no, is there anything about ECD that you are not yet convinced about?**

11. Think about your favorite ECD session you have attended. What was good about it?

***PROBE*** *(****ask if they like it this way****):*

*What was the session about?*

*Was it at the facility or in the community?*

*Were there other mothers in the session or was it only you and the Expert Client?*

*How long was the session?*

12. We know the program is not perfect and we would like your help to improve it. Think about your LEAST favorite ECD session you have attended. What did you NOT like?

***PROBE (ask if they like it this way,***

***If NO least favorite mentioned, ask about other mothers):***

*What was the session about?*

*Was it at the facility or in the community?*

*Were there other mothers in the session or was it only you and the Expert Client?*

*How long was the session?*

*What would have made the meeting better?*

1. Think about the last ECD session you attended. How long did the session last?

***PROBE:*** *Would you like more or less time? Why?*

1. Most of the times people in the same program know each other and even talk to each other outside the facility. With your fellow women in the program do you communicate outside the facility like in the village?

***Probe: If Yes:*** *What do you talk about? Do you share topics learnt when someone missed a session? Do these friends help you in anyway? How?*

***Probe: If No;*** *why not?*

**Section 3: Impact of ECD sessions**

Now I’d like to understand if you think the ECD program has changed anything for you and your youngest child.

1. What has changed because of the ECD program? (*Give specific examples of how things were before the ECD program compared to after the program)*
2. Has the program changed anything between you and your child?
3. Between you and your husband (or the child’s primary male caregiver)?
4. Between the husband/ male caregiver and the child?
5. Has the program changed your experience at the health facility, in terms of time, the way facility staff treat you, ART services you receive?

***Probe: if nothing has changed****: what do you like about your experience at the facility in terms of how you are treated etc.*

**Section 4: Unmet Needs Remaining**

1. What could be done differently in the ECD Program to help you better?
2. Apart from the early childhood development program, is there anything in the MIP program that can be changed or made better?

**Section 5: Barriers and Facilitators**

We have talked about what you know about ECD and why you are motivated to practice ECD at home, but there are other things happening at home or in your life that might affect how much you practice ECD at home. I would like to know about some of those things

1. What do you think enables you to practice ECD with your youngest child at home? (either from ECD Program or something else)

***PROBE****: How?*

1. We understand that sometimes mothers might not be able to practice ECD at home as much as they would want to. What are some challenges you have to overcome to practice ECD with your child/children at home?

***PROBE, if none:*** *Think about other mothers in your community. What are some challenges to practicing ECD that they might face?*

**Section 6: Suggestions for Engaging Male Partners**

Thank you for all the information. We would like to make ECD programs relevant to your male care givers partners as well, where men are interested in attending meetings and engaging in ECD activities. I would like to know your opinion about your male care giver of your youngest child in order to help us better engage men in ECD.

**Childcare**

1. **(*Skip this question if mother has already identified a male caregiver*)** Think about the man who acts most like a father for your youngest child. Who is this man?

***PROBE:*** *Father, husband, uncle, grandfather*

**(If there is no father figure at all, Skip to Q23, Q29, Q30 and Q31**

1. In an average week, how much time does your MALE CAREGIVER spend with your youngest child? What does he usually do with the child?

***PROBE:*** *Holding, feeding, playing?*

***PROBE, If NOT involved in childcare:*** *Why is he not involved (what are barriers)? What could make him be more involved in childcare?*

1. Are there specific tasks for your youngest child that MALE CAREGIVER is responsible for? Please describe each one.

***PROBE:*** *feeding, providing food, providing clothes (etc.)*

***PROBE:*** *are there any other tasks that you’d like MALE CAREGIVERS to be responsible for or help with?*

1. From your point of view, what should a good father do for ECD?
2. Now that you have mentioned that, do you think the MALE CARE GIVER is a good father as far as ECD is concerned:

***PROBE:*** *Why/ why not?*

1. What ECD activity do you think MALE CAREGIVER likes the most?

**ECD Program**

1. **(*Skip this question if it has already been answered*)** *MALE CAREGIVER* is aware of the program?

***PROBE, NO:*** *Why is he not aware?*

***PROBE IF YES:*** *What does he know about the program?*

1. Do you talk about the program with *MALE CAREGIVER*?

***PROBE, IF YES:*** *What do you usually tell him? Do you share the lessons with him?*

***PROBE:*** *What does he think about the program? How does he feel about it?*

***PROBE:*** *Does he think ECD for infants is important?*

***PROBE:*** *Does he try to practice ECD at home with your youngest child?*

Thank you for sharing all of this information. We want to set up this program in a way that includes your fathers and father figures and encourages them to come. We know it is not easy for men to come to the clinic, but we believe it is important to engage male caregivers in ECD, so we would like get some recommendations about how we can do this.

1. Would your MALE CAREGIVER feel comfortable coming to the ART clinic for this program?
2. What about other men in your community? Would they feel comfortable.

***PROBE:*** *What do you think is the main reason he/they cannot feel comfortable coming to the ART clinic?*

1. Do you think he/they would attend an ECD session with you/their spouses if it were on a non-ART/MIP day?
2. Do you think he/they would attend an ECD session on a non-ART/MIP day if only men were included?
3. Is there anything else that would encourage him to come to the ECD sessions?
4. In your opinion, what can the ECD program do to be more welcoming to fathers?
5. What do you think is the best way to help encourage [MALE CAREGIVER] to do ECD activities at home?
6. Would [MALE CAREGIVER] be interested in getting a pamphlet about ECD with pictures that are easy to understand?
   1. PROBE, YES: *Do you think a pamphlet would help him practice more ECD activities?*

**(Thank the respondent and end the interview)**
